# Built differently or defective: can RNA exosomopathies cause ribosome heterogeneity?

**DOI:** 10.1098/rstb.2023.0382

**Published:** 2025-03-06

**Authors:** Zachary J. Bressman, Anita H. Corbett, Homa Ghalei

**Affiliations:** ^1^ Department of Biochemistry, Emory University School of Medicine, Atlanta, GA 30322, USA; ^2^ Department of Biology, Emory College of Arts and Sciences, Atlanta, GA 30322, USA; ^3^ Graduate Program in Biochemistry, Cell, and Developmental Biology, Emory University, Atlanta, GA 30322, USA

**Keywords:** RNA exosome, ribosome, RNA exosomopathies, ribosome heterogeneity, RNA processing, translation

## Abstract

The RNA exosome is an essential, evolutionarily conserved ribonuclease required for processing and degradation of key cellular RNAs. The complex maintains RNA homeostasis within every cell by ensuring the proper maturation, quality control and turnover of various RNA species including rRNAs. A growing list of diseases, collectively termed RNA exosomopathies, are caused by mutations in genes encoding structural subunits of the RNA exosome complex. RNA exosomopathies often result in tissue-specific defects, particularly manifesting as neurological disorders, which is intriguing given the ubiquitous functions and expression of the RNA exosome. One such ubiquitous, essential function of the RNA exosome is its involvement in ribosome biogenesis. In this review, we discuss the established connections between the RNA exosome and ribosome biogenesis, exploring the potential mechanisms through which RNA exosomopathies could influence ribosome heterogeneity, leading to aberrant translation and pathogenesis. We highlight the critical need for research in this area that can aid in understanding the complex aetiology of RNA exosomopathies and the future development of therapeutic strategies to mitigate pathology.

This article is part of the discussion meeting issue ‘Ribosome diversity and its impact on protein synthesis, development and disease’

## 1. Introduction

The RNA exosome is a ubiquitous, essential, evolutionarily conserved 3′ to 5′ exo/endoribonuclease that is required for processing and degradation of cellular RNAs both in the nucleus and the cytoplasm [1–3]. The RNA exosome complex consists of a three-subunit cap and a six-subunit barrel-shaped core that are attached to a catalytic base [4–8]. The RNA substrates of the complex are diverse and include all major cellular RNA classes ranging from messenger RNAs (mRNAs), ribosomal RNAs (rRNAs) and transfer RNAs (tRNAs) to small and large non-coding RNAs (ncRNAs; [1,9–13]). A growing number of pathogenic amino acid variants in the structural subunits of RNA exosomes are linked to diseases, which have been termed RNA exosomopathies [14–16]. Despite the ubiquitous role of the RNA exosome in all cell types, RNA exosomopathies often cause tissue-specific defects. Pathogenic missense mutations have been identified in nearly all RNA exosome subunits (table 1; [17–24,27–29]). Missense mutations in the genes encoding the cap subunits, EXOSC1 and EXOSC3, and the core subunits, EXOSC8 and EXOSC9, cause forms of a severe neurodevelopmental disorder, pontocerebellar hypoplasia [17,18,20,23,24,27–30]. Missense mutations in the *EXOSC2* cap subunit gene lead to a novel syndrome characterized by short stature, hearing loss, retinitis pigmentosa and , which has been termed SHRF [19,31]. A biallelic variant of the core *EXOSC4* gene causes a neurodevelopmental disorder with brain atrophy and progressive postnatal growth retardation [21]. Finally, pathogenic mutations in the gene encoding the core EXOSC5 subunit are associated with a disease characterized by cerebellar atrophy, spinal muscular atrophy-like motor delays and hypotonia [22]. This collection of diseases with varying pathology highlights the critical role of the RNA exosome in maintaining cellular RNA integrity and the profound impact of single amino acid changes in the RNA exosome complex on clinical presentation.

**Table 1.** RNA exosome subunits and their disease association.

	human gene name	*Saccharomyces cerevisiae* gene name	disease association
**cap subunit**	*EXOSC1*	*CSL4*	pontocerebellar hypoplasia (PCH; [17,18])
	*EXOSC2*	*RRP4*	short stature, hearing loss, retinitis pigmentosa, distinctive facies (SHRF; [19])
	*EXOSC3*	*RRP40*	PCH type 1b or cerebellar hypoplasia [20]
**core subunit**	*EXOSC4*	*RRP41*	unclassified disease with symptoms that include heavily impaired mobility, cognitive impairment, iron deficiency anaemia, axial hypotonia and more [21]
	*EXOSC5*	*RRP46*	cerebellar hypoplasia [22] additional features include: failure to thrive, short stature, feeding difficulties, developmental delays that lead to motor deficiencies, hypotonia and esotropia
	*EXOSC6*	*MTR3*	none reported
	*EXOSC7*	*RRP42*	none reported
	*EXOSC8*	*RRP43*	PCH type 1c [23]
	*EXOSC9*	*RRP45*	PCH type 1d [24]
**catalytic subunit**	*DIS3*	*RRP44*	multiple myeloma [25] myeloid leukaemia [26]

Table 1 summarizes the list of RNA exosome subunits in budding yeast and humans and the diseases associated with each subunit. Due to the functional and structural conservation of the RNA exosome complex, *Saccharomyces cerevisiae* (budding yeast) has proven to be a powerful model organism for investigation of the fundamental molecular defects arising from RNA exosomopathy-linked variants [15,22,32–35]. Recent evidence from studies in budding yeast strongly suggests that RNA exosomopathy variants influence ribosome biogenesis and heterogeneity [21,35]. In this review, we discuss how pathogenic RNA exosome variants could result in production of defective or heterogeneous ribosomes. We focus on the ribosome components, i.e. ribosomal proteins (RPs) and rRNAs, to highlight the potential direct effects on the ribosome rather than ribosome interacting and accessory factors, or signalling pathways that regulate the process or potential genomic and transcriptomic changes that may arise as a result of RNA exosomopathy mutants. We discuss potential major scenarios for how heterogeneous ribosomes could be generated in RNA exosomopathies, by directly affecting the ribosome components, and propose future directions for the study of RNA exosome variants and their relationship to translation.

### (a) Can RNA exosomopathies cause ribosomal RNA heterogeneity?

Ribosomal RNAs account for over 80% of the transcriptome mass [36]. The small ribosomal subunit (40S) is composed of 18S rRNA and the large ribosomal subunit (60S) is composed of 28S (25S in budding yeast), 5.8S and 5S rRNAs. The mature rRNAs are processed from precursor transcripts through a highly intricate and regulated pathway involving the action of several nucleases including the RNA exosome [37,38]. The RNA exosome plays a critical role in structural rearrangements of the small subunit processome required for maturation of 40S subunits [39,40]. The RNA exosome is also required in a multi-step process of trimming the precursor 7S rRNA transcript for maturation of 5.8S rRNA during biogenesis of the 60S subunit [41,42].

Several studies of the RNA exosomopathy variants have confirmed a loss of quality control in rRNA processing, marked by accumulation of 7S rRNA and reduction of 5.8S rRNA [21,22,34,35,43]. In agreement with the ribosome biogenesis defects, expression of the cap or core RNA exosomopathy variants as the sole copy of the essential RNA exosome subunit in yeast results in reduced levels of polysomes [21,35]. Northern blot analysis of polysome profiles of yeast cells modelling RNA exosomopathy-linked variants shows that a fraction of the accumulated 7S pre-rRNA enters the translation pool, producing a heterogeneous pool of translating ribosomes [21,35]. The basis for this statement relies on experiments where cycloheximide is omitted from the gradients resulting in runoff of translating ribosomes, combined with the addition of puromycin, which blocks nascent polypeptide chain elongation, thereby terminating translation and resulting in disassociation of the ribosomal subunits. Results of these studies show that these 7S-containing particles are not aggregates or heavy complexes that co-sediment with polysomes [21,35].

Previous studies have described 7S-containing ribosomes that enter the translation pool in *mtr4-(dob1−1)* mutant yeast cells [44]. Mtr4 is an evolutionarily conserved RNA helicase that acts as a co-factor for the RNA exosome [45]. Structural analysis of the nuclear RNA exosome bound to pre-ribosome has revealed that Mtr4 is a major contact for attaching the RNA exosome complex onto pre-ribosomes for 3′-end trimming of 5.8S rRNA [46]. Interestingly, the interaction of Mtr4 with the RNA exosome is weakened in at least one yeast RNA exosomopathy model [43]. Thus, beyond changes in the integrity or cellular levels of the RNA exosome in RNA exosomopathies that can impact rRNA processing, changes in the interactome of the complex can also influence ribosome biogenesis and contribute to ribosome heterogeneity.

In addition to the critical roles of the RNA exosome during biogenesis of small and large ribosomal subunits, the complex is required for maturation of small nucleolar RNAs (snoRNAs), an abundant group of ncRNAs critical for proper rRNA processing and cleavage as well as guiding the majority of rRNA chemical modifications [47–49]. snoRNAs are also required for chemical modification of small nuclear RNAs (snRNAs), which are critical components of the spliceosome required for proper assembly and function [50]. Deregulation of snoRNAs, snoRNA biogenesis or snoRNA-guided chemical modifications is implicated in many human diseases [49,51–55]. While the majority of snoRNAs are ubiquitously expressed in different tissues, some have tissue-specific enrichment in the brain [56,57] and reproductive tissues [57]. Unlike uniformly expressed snoRNAs [58], snoRNAs with tissue-specificity are primarily embedded in long noncoding RNAs (lncRNAs) and the majority of them are orphans with no currently defined targets [59]. snoRNAs can also function by acting in *cis* to regulate the expression of their host genes, including translation factors, or result in production of composite extended transcripts with putative roles that may contribute to pathogenicity when deregulated [60]. Because the RNA exosome complex is critical for snoRNA biogenesis, RNA exosomopathies may therefore also contribute to deregulation of snoRNAs and their downstream targets including snRNAs. In the context of ribosome biogenesis, while the RNA exosome and other ribonucleases are essential for cleavage steps during ribosome biogenesis, snoRNAs are critical for chaperoning rRNA folding and key processing events, as well as rRNA chemical modifications, which can add another layer of heterogeneity to ribosomes [61]. Because the RNA exosome plays a crucial role in biogenesis of mature and functional snoRNAs, changes in the amount, activity or interactome of RNA exosome as a result of RNA exosomopathy variants can therefore cause changes in the steady-state cellular levels of snoRNAs [1,13]. These alterations, in turn, may affect rRNA modification patterns that tune translation [62]. Because the RNA exosome is required for maturation of the majority of snoRNAs [48,49], a global change in steady-state snoRNA levels would likely affect rRNA modification sites at several variable positions within rRNA transcripts, as has been observed when snoRNP biogenesis is impaired in budding yeast models [63,64]. A site-specific large-scale change of variable rRNA modifications can cause ribosome biogenesis defects [64,65], alter the dynamics of ribosomes required for translocation, impact translation fidelity and impair the interaction of ribosomes with translation factors [63]. Changes in snoRNA levels or rRNA modification may also impact the mRNA preference of ribosomes. For example, cells lacking snoRD45C, which guides 18 S-Cm174 modification, show codon-specific changes in translation [66]. In addition, because several snoRNAs are expressed in a tissue-specific manner, including some involved in rRNA modification [57], dysregulated levels of snoRNAs could exert differential impacts on translation in different cell types, in line with tissue-specific defects arising in RNA exosomopathies.

In summary, changes in rRNA processing and modification that arise from RNA exosomopathies have the potential to cause ribosome heterogeneity that may contribute to the compound pathology of these diseases. Future biochemical analysis and functional assays of RNA exosomopathy variants will be critical to elucidate how each variant mechanistically impacts ribosome biogenesis, whether there are common themes across variants in different subunits, and finally to what extent and how the heterogeneity of the translation pool with a fraction of ribosomes containing misprocessed rRNAs can impact translation in RNA exosomopathies.

### (b) Can RNA exosomopathies cause ribosomal protein heterogeneity?

In addition to rRNAs, the small ribosomal subunit contains 33 RPs and the large subunit contains 49 RPs. RP mRNAs are among the top differentially expressed mRNAs in RNA exosomopathy models studied to date [30,35]. The assembly of RPs into ribosomal subunits follows a highly regulated and orchestrated series of steps to ensure the timely and accurate incorporation of each RP [67]. Imbalanced RP levels in RNA exosomopathies may, therefore, result in deregulated ribosome assembly. Indeed, haploinsufficiency of RPs in ribosomopathies results in ribosome biogenesis defects and reduced ribosome levels [68]. Reduced RP levels in RNA exosomopathies may affect rRNA processing, cause changes in ribosome composition due to failure in proper RP incorporation, or a combination of both effects. RNA-exosomopathy mutants may also impact the expression of RP paralogues by directly impacting their specific mRNAs or indirectly affecting the regulatory genetic crosstalk between RP paralogues [69,70]. A few RPs in human cells have paralogues, some with tissue-specific expression [71–73], which could contribute to ribosome specialization. Any of these scenarios could result in a significant reduction in the available pool of ‘regular’ translating ribosomes or ribosome heterogeneity, causing significant changes to translational output of the cell and potentially resulting in mRNA-specific outcomes [74–78]. In addition, release of premature ribosomes into the translation pool [79–81], decay of translating ribosomes [80] or ribosome repair [82] could contribute to ribosome heterogeneity when RPs are impacted. These changes can have widespread implications for cellular function, as both fewer ribosomes and ribosomes with altered compositions can impact the translational output [83].

Similar to rRNAs that are chemically modified, several RPs undergo post-translational modifications (PTMs) including phosphorylation, methylation, acetylation and hydroxylation, among others [84–92]. Some of these PTMs are critical for translation regulation [84,86,93]. Deregulated PTM of ribosomes is associated with disease. For example, ribosomes undergo hyperphosphorylation in dysmorphism [94] and Parkinson’s disease [95]. Variations to the RNA exosome as a result of RNA exosomopathies could alter the expression of genes required for deposition of PTMs. Such a change could impact RP stability, assembly into the ribosome or function, resulting in changes in overall cellular translation.

Overall, RNA exosomopathies could result in changes in RP levels, to the ratio of RP paralogues, defective incorporation of RPs into ribosomes, altered PTMs or a combination of these scenarios, all of which could cause ribosome heterogeneity contributing to disease pathology. Future studies to establish whether and how RNA exosomopathies can affect RP composition of the translating pool of ribosomes will be critical to reveal the molecular changes arising from RNA exosomopathy variants.

## 2. Conclusions and future directions

**Figure 1.**
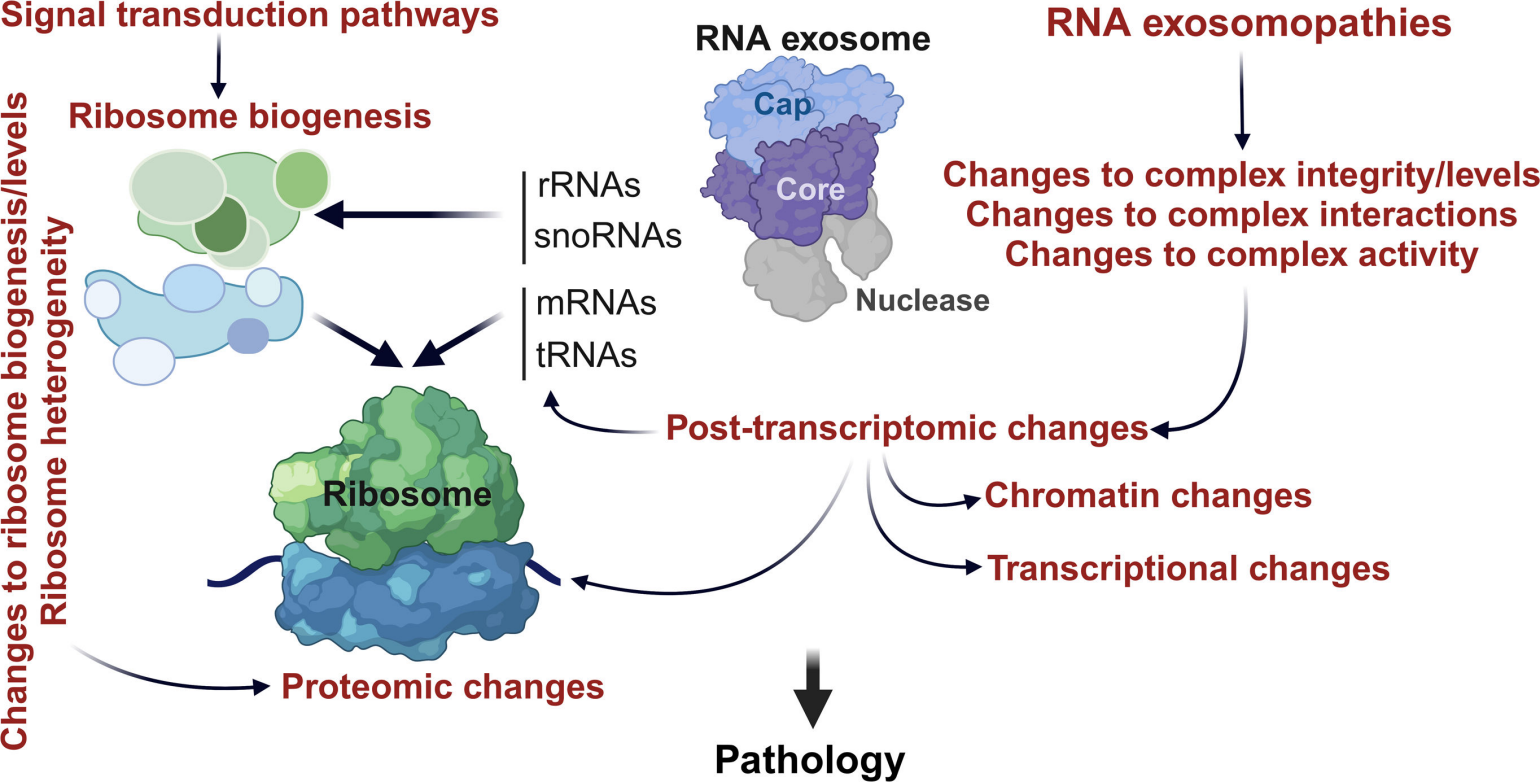
Overview of how RNA exosomopathy variants can cause pathogenicity by impacting ribosomes. Pathogenic missense mutations in the structural subunits of the RNA exosome can result in changes in complex integrity and steady-state levels, the interactome of the complex and/or the activity of the complex. These changes in turn can result in a wide range of post-transcriptomic alterations affecting the steady-state levels of many RNAs required for ribosome biogenesis and translation, as well as RNAs required for chromatin regulation, production of transcription factors or signal transduction proteins. In the context of mature ribosomes, incorporation of aberrant rRNAs, changes in rRNA modification pattern, changes to ribosomal protein stoichiometry, ribosomal protein paralogues and/or post-translational modifications can directly impact ribosome concentration and composition. Created with BioRender.com

While the list of RNA exosomopathy variants is growing, the molecular mechanisms of the diverse outcomes of these diseases remain poorly understood. Because the RNA exosome is involved in many different RNA processing pathways, there could be many functional consequences of a missense mutation in a gene encoding any of the RNA exosome subunits any of which could directly or indirectly affect ribosomes (figure 1). Because rRNA accounts for a significant portion of the transcriptional output and due to the major role of the RNA exosome in biogenesis of both ribosomal subunits and the large impact of translational changes on cellular outcomes, studies of the precise molecular impacts of RNA exosomopathy variants on ribosomes are critically important to understand the major molecular drivers of RNA exosomopathies. Recent evidence suggests that aberrant ribosomes are made and participate in translation in budding yeast RNA exosomopathy models [21,35]. This finding emphasizes the need to move the functional studies of RNA exosomopathies beyond the RNA exosome itself, and points to heterogenous ribosomes as another potential molecular effector in RNA exosomopathies.

Ribosomes interact with a variety of factors throughout their life. Many of these interactions are required for quality control to ensure the proper production and function of ribosomes. The RNA exosome plays a key role in quality control of ribosomes during their biogenesis. Lack of this quality control leads to the accumulation of aberrant precursor rRNAs observed in RNA exosomopathy models [21,22,35]. Defective pre-rRNAs may accumulate as they cannot be efficiently degraded, which can lead to production of defective or heterogeneous ribosomes. The RNA exosome function is also essential for productive export of pre-ribosomes from the nucleus to the cytoplasm [96], which can also be impacted in RNA exosomopathies, depleting the pool of functional cytoplasmic ribosomes that can engage in translation and impacting cells through lowering cytoplasmic pools of ribosomes. While there is currently evidence that 7S-containing ribosomes in budding yeast can enter the translation pool, there is no data to demonstrate whether these ribosomes impact protein synthesis. Whether 7S-containing ribosomes that enter the translation pool actively engage in translation or are eventually cleared out by other quality control mechanisms is not yet known. For example, these 7S-containing ribosomes may be identified by ribosome collisions when they encounter normal ribosomes resulting in their degradation [80]. Future work will be required to understand how the 7S-rRNA-containing ribosomes do or do not alter the proteome.

Defects in ribosome biogenesis cause human diseases that share similarities with RNA exosomopathies. Both ribosomopathies and RNA exosomopathies cause tissue-specific defects despite being caused by changes in ubiquitous cellular machines that are required for the function of every cell. Neurological impairment and neurodevelopmental delays are common phenotypes across several ribosomopathies and RNA exosomopathies [14,68]. Why mutations in genes encoding structural subunits of the RNA exosome are more consequential in some specific cell types or tissues, such as specific neuronal populations, remains unclear to date. Despite the notion that RNA exosomopathies are pathogenic situations that can result in lower ribosome levels and production of heterogenous or defective ribosomes, containing an abnormally processed rRNA, these ribosomes support life. Defining the molecular mechanisms by which these different or defective ribosomes contribute to heterogeneity of the translation pool and impact the cellular proteome is crucial to define molecular mechanisms of RNA exosomopathies.

A challenge in studies of RNA exosomopathies remains to distinguish the causative effects from secondary effects due to the wide range of the substrates targeted by this complex. The ribosome biogenesis pathway is tightly regulated, internally by quality control checkpoints [97] and externally through signal transduction pathways [98]. RNA exosomopathies can lead to temporal regulation of translation in cells to selectively upregulate the pathways necessary for survival. For example, defects in ribosome biogenesis result in the stabilization of the pro-apoptotic stress-induced transcription factor p53, causing apoptosis and cell cycle arrest [98,99]. Suppression of p53 signalling by RNA exosome has been reported to be critical for normal cell survival and brain development [100]. In line with this report, p53 mRNA levels are increased in zebrafish models of RNA exosomopathies, and the mutant zebrafish have brain developmental defects due to increased cellular apoptosis [30]. Thus, changes in translation in RNA exosomopathies are also likely influenced by the signalling pathways that regulate and are regulated by translation [98].

In the context of translating ribosomes, distinguishing defective ribosomes en route to degradation versus different or specialized ribosomes, mixed with canonical ribosomes in the translation pool, contributing to cell survival, will be critical. The combined power of *in vivo* studies with *in vitro* ribosome biochemistry, structural biology and functional assays could be a powerful approach to pin down the contribution of variations of the ribosome to pathology that occurs in RNA exosomopathies. Such studies can not only reveal how altered RNA exosome function impacts cellular translation but may also provide new insights into potential therapeutic approaches.

## Data Availability

This article has no additional data.
